# Establishing Monitored Premises Status for Continuity of Business Permits During an HPAI Outbreak

**DOI:** 10.3389/fvets.2018.00129

**Published:** 2018-06-22

**Authors:** Jamie Umber, Rebecca Johnson, Michelle Kromm, Eric Linskens, Marie R. Culhane, Timothy Goldsmith, David Halvorson, Carol Cardona

**Affiliations:** ^1^Secure Food Systems Team, University of Minnesota Twin Cities, Saint Paul, MN, United States; ^2^Department of Veterinary and Biomedical Sciences, University of Minnesota, St. Paul, MN, United States; ^3^Jennie-O Turkey Store, Willmar, MN, United States; ^4^Department of Veterinary Population Medicine, University of Minnesota, St. Paul, MN, United States

**Keywords:** HPAI, disease outbreaks, monitored premises, continuity of business, permit, permitted movement, questionnaire

## Abstract

Recent experiences with avian influenza outbreaks in poultry in the United States have tested biosecurity protocols and outbreak management strategies. During an outbreak, regulatory officials managing the emergency response need to make timely decisions in order to achieve disease control and eradication goals while simultaneously decreasing the unintended consequences of the response. To move susceptible animals or animal products out of a disease Control Area via a secure food supply continuity of business (COB) permit without the risk of expanding a disease outbreak, premises must be designated as Monitored Premises (MP) by regulatory officials. The experience of and lessons learned from the 2014 to 2015 highly pathogenic avian influenza (HPAI) outbreak have resulted in defined criteria necessary to establish MP status during an HPAI outbreak and highlighted the need for a clear method to determine that those criteria have been met. Establishing MP status is different from an epidemiologic investigation, though they both require analyses of how avian influenza virus may enter poultry premises and can take significant staff time. MP status of premises seeking to move animals or animal products must be continuously re-evaluated as Infected Premises status, and resulting epidemiologic contacts, can rapidly change during an outbreak. We present here a questionnaire to establish MP status, designed to be initially completed by industry representatives in an attempt to streamline processes and conserve resources. During an outbreak, the MP status questionnaire is an essential risk-based management tool used to establish premises status, as part of operationalizing permitted movement to support COB.

## Introduction

The process for moving animals and animal products in the United States (US) can be challenging to implement when quarantine and movement control activities are in place to contain and eradicate a foreign animal disease (FAD) such as highly pathogenic avian influenza (HPAI). However, facilitating the movement of non-infected animals and non-contaminated animal products into, within, and out of a disease Control Area during a disease outbreak, while minimizing risk of disease introduction and/or spread, is critical in order to maintain continuity of business (COB) for animal agricultural industries (1, 2). For poultry, COB is achieved when the movement of non-infected birds and non-contaminated poultry products are allowed during an HPAI outbreak, thus helping prevent many potentially devastating unintended economic consequences of the outbreak and securing the US food supply. Additionally and importantly, though outside the scope of this manuscript, maintaining COB helps address animal welfare issues that can arise due to restricted movements.

## Federal guidelines and definitions

**Figure 1:**
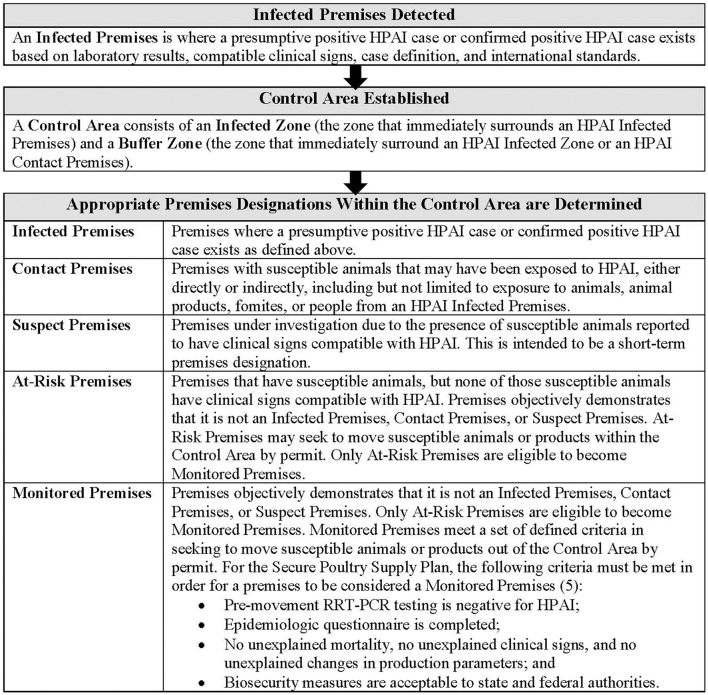
USDA APHIS and Secure Poultry Supply Plan definitions of and steps leading to HPAI area, zone, and premises designations. Additional FAD zone, area, and premises designation definitions not applicable to this manuscript are available in The Red Book (3). Within the unified Incident Command*, the Incident Commander works with the Operations Section and Situation Unit (in the Planning Section) to determine zone, area, and premises designations. These designations are evaluated and reevaluated as needed throughout an outbreak based on the epidemiological situation; specific guidelines as to how to and who will conduct such evaluations to determine appropriate premises designations do not exist. The MPSQ provides a method to establish Monitored Premises status. *In the US, HPAI response is based on the principles found in the National Response Framework (NRF) and National Incident Management System (NIMS); response efforts should be implemented through a Unified Incident Command (i.e., in a manner consistent with the Incident Command System [ICS]) (see the HPAI Red Book for more detailed information and references for the Unified Incident Command organizational structure).

“The United States Department of Agriculture Animal and Plant Health Inspection Service (USDA APHIS) HPAI Response Plan: The Red Book” is the federal document detailing the FAD Preparedness and Response Plan to HPAI in the United States. The plan stipulates that when HPAI is detected in the US, appropriate regulatory officials issue a quarantine, hold order, or standstill notice for the Infected Premises and establish the boundaries of a Control Area (3). Regulatory officials also work to determine appropriate premises designations (i.e., Infected, Contact, Suspect, At-Risk, and Monitored) for other poultry operations within that Control Area (Figure 1). The area and premises designations are used for quarantine and movement control efforts, which extend beyond the Infected Premises and are implemented as rapidly as possible [see also Figure 5-4 in The Red Book for a graphic representation of premises designations in relation to permitting and movement control (3)]. Quarantine and movement restrictions are important tools in controlling and eradicating any FAD outbreak; however, there remain multiple types of movements that occur during an FAD outbreak that are critical to the vitality of the animal agriculture business and which can be done with minimal risk of spreading disease (e.g., movements of feed, liquid pasteurized egg products, processed meat, or newly hatched birds). So as not to create an unacceptable risk of disease spread, the current US approach to HPAI emergency response involves regulatory officials issuing permits for some movements to, from, and within Control Areas [e.g., from movements of susceptible animals and animal products to movements of fomites and materials (4)]. Commodity-specific proactive risk assessments help inform the permit decision-making processes with regard to which types of movements from apparently healthy animals (flocks or herds) may pose acceptable risk.

According to federal guidelines, there are two primary types of permits: (1) Specific, and (2) COB (4). Specific permits are used for movements from Infected, Contact, and Suspect Premises—which are under quarantine during an FAD outbreak. COB permits may be issued for movements from At-Risk Premises or Monitored Premises (MP) and are vital for the production of animals and animal products (4). COB permits are meant to facilitate the continuation of business operations for those premises not infected by the disease agent but still affected by their location within a Control Area and the associated movement restrictions therein. Secure Food Supply (SFS) permits, a type of COB permit, allow movements of animals and animal products into the supply chain for further feeding, growing, processing, or to market (4). At-Risk Premises may seek a COB SFS permit to move susceptible animals or animal products *within* the Control Area. Monitored Premises (MP) meet a set of defined criteria and may seek to move susceptible animals or animal products both within and out of the Control Area by COB SFS permit. Movements into the Control Area under COB SFS permits are less common (4). An MP objectively demonstrates that it is not an Infected Premises, Contact Premises, or Suspect Premises.

Our focus here is on COB SFS permitted movements from MP [for further information about other movement or permit types see the FAD Preparedness and Response Manual, Permitted Movement (4)]. It is important to note that outbreak-specific circumstances cannot be predicted in advance and therefore movement permitting decisions may ultimately depend on relevant risk and epidemiologic determinations made by regulatory officials for any given outbreak.

## Background

The goals of HPAI response in the US include eradicating HPAI (using strategies that stabilize animal agriculture, the food supply, and the economy while protecting public health and the environment) and providing science- and risk-based approaches and systems to facilitate COB (3). Achieving these goals will allow US industries to resume normal production as quickly as possible and the US to regain disease-free status, ideally without the response effort causing more disruption and damage than the disease outbreak itself (3) (e.g., since avian infection with AI viruses is notifiable to the World Organisation for Animal Health [or OIE] there may be significant international trade consequences until disease-free status can be regained). With these goals in mind, the concepts and definitions for premises designations have evolved with FAD preparedness and response in the US. The term “Monitored Premises” first appeared in the USDA HPAI Response Plan (i.e., The Red Book) in 2011. Only At-Risk Premises are eligible to become MP (i.e., currently Infected, Contact, and Suspect Premises cannot become Monitored Premises) (3). According to the 2017 Red Book, MP meet a set of defined criteria in seeking to move susceptible birds or poultry products out of the HPAI Control Area by permit; these criteria are based on the level of risk of the movement and are set out by the Secure Poultry Supply Plan.^1^ For the Secure Poultry Supply Plan, the following criteria must be met in order for a premises to be considered an MP [the combined USDA APHIS and Secure Poultry Supply Plan definition of MP is included in Figure 1; (3, 5)]:

- Pre-movement RRT-PCR testing is negative for HPAI;
- Epidemiologic questionnaire is completed;
- No unexplained mortality, no unexplained clinical signs, and no unexplained changes in production parameters; and
- Biosecurity measures are acceptable to state and federal authorities.

The criteria specified in the Secure Poultry Supply Plan MP definition were harmonized across poultry industries based, in part, on observations during the 2014–2015 HPAI outbreak in the US and on consultations with the Secure Poultry Supply Working Groups representing the egg, turkey, and broiler industries. While these overarching criteria were harmonized, details regarding each component may be industry specific. Numbers and timing of samples collected for RRT-PCR testing may differ by industry based on transmission rates, products being moved, and nature of the production system, for example. Both the Secure Egg Supply and the Secure Turkey Supply plans include epidemiology questionnaires (which are tailored to identify any possible source of HPAI retrospectively and prospectively on egg or turkey operations, respectively), while the Secure Broiler Supply plan does not.

Lessons learned regarding permitted movement during the 2014–2015 HPAI outbreak in Minnesota (MN) were the subject of several cross-sector/cross-commodity meetings of individuals from the poultry industry, academia, and state and federal agencies that began in late 2015 with a goal to improve future permitting. These meetings comprised larger group discussions as well as smaller working group discussions. The first multi-disciplinary meeting was held by the University of MN (UMN) in December 2015, and it highlighted how permitted movement must be a collaborative effort and resulted in the formation a working group charged with creating a revamped permitting process for MN (6). A key question that this permitting working group faced was how to establish MP status (i.e., how to determine that all necessary criteria had been met) and who will do it.

## Development of a tool to establish monitored premises status

Prior to the 2014–2015 HPAI outbreak in the US, permit guidance in MN generally was written *ad hoc* (to address biosecurity and testing requirements) for use by regulatory officials to issue movement permits for poultry. Regulatory officials would review basic production parameters and epidemiologic links among poultry farms that requested to move birds or poultry products. During the 2014–2015 HPAI outbreak in MN, the permitting language was long and very product-specific, all permits were hand-signed, and initially trucks were officially sealed and followed to destinations such as processing plants. This permitting process created a significant workload burden on regulatory officials during the outbreak, requiring an average of 288 staff hours per week (equivalent to 7 full-time employees) for 16 weeks, not including federal staff or indirect state staff time. In the end, over 900 permits were approved in MN that encompassed over 3,000 movements (not including feed or slaughter product permits and their associated movements) (7).

While the permitting process in MN ultimately was successful during the 2014–2015 HPAI outbreak, the substantial time and effort required by regulatory officials and industry alike for permitting was sufficient to motivate an improvement of the process. According to the USDA, during the entire 2014–2015 HPAI outbreak (which involved 15 states that had positive commercial or backyard poultry producing premises), there were over 7,500 permits—and over 20,000 individual movements associated with those permits—that were entered into the USDA APHIS Emergency Management Response System 2.0 (EMRS2). EMRS2, a secure information management system, is used by APHIS personnel for all permitting processes, including issuing permits and tracking movements (4). While a large portion (36%) of the total permits during the outbreak were issued in MN (8), given the sheer number of permits involved for the US as a whole, the burden of the permitting process is likely to have been a challenge for other states as well, not just MN.

The cross-sector/cross-commodity meetings held in MN following the 2015 outbreak were an opportunity to garner the expertise of people involved in this very large outbreak on the aspects of response that went well and aspects that needed improvement. Through multiple meetings and conversations, it became clear that one key component of the permitting process was lacking—a clear method for establishing MP status. Thus, developing a procedure and the tools to establish that all necessary criteria for MP status have been met could help improve the permitting process in future disease outbreaks. Determining MP status is different from an epidemiologic investigation, making epidemiologic questionnaires inefficient tools for determining MP status. When determining MP status, it is important to elicit evidence of potential infection via contact specifically with Infected, Suspect, or Contact Premises in a Control Area. In contrast, epidemiologic investigations are more open-ended and examine contact with *all* potential sources (including other poultry operations and wild birds) and may also seek to gather information about a premises that is not central to determining MP status, such as management type. As such, having a targeted, pre-existing “Monitored Premises Status Questionnaire” (MPSQ) could assist regulatory officials and industry representatives when they are seeking to move poultry/poultry products via COB SFS permits. As the MN working group began development of the MN MPSQ, the 2016 AI outbreak in Indiana re-confirmed the need for such a tool. The response coordinators in the 2016 Indiana outbreak benefited from lessons learned and tools developed as a result of the 2014–2015 outbreak; however, no pre-existing method for determining MP status had been developed at that time. The Indiana Incident Command created their own impromptu strategy during the outbreak. Their method involved using direct communications with the poultry industry premises, completing a biosecurity checklist (using the existing biosecurity checklist in the Secure Egg Supply Plan), and conducting daily sampling (personal communication Dr. Mike Kopp, Sept 2017).

It follows that a pre-existing MPSQ should enable more efficient determination of the appropriate premises designation, that is, if a premises meets the defined criteria to be designated as an MP. This determination would ideally be made without having to have as many direct conversations or searching for then modifying existing checklists or questionnaires to address all of the defined criteria. A set of targeted questions was thus compiled to create an MPSQ that, taken together, industry could use to establish whether a premises had met all the necessary criteria for MP status (Table 1). The MPSQ is divided into four sections: Identification & Location of Premises; HPAI; Epidemiologic Links/Exposures to Infected Premises; and Biosecurity. The Identification & Location of Premises section identifies the premises via the national Premises Identification Number. The HPAI section contains questions to help determine whether or not a premises is an Infected Premises or a Suspect Premises (e.g., does the premise have a diagnosis of HPAI or does the premises have any unexplained clinical signs or clinical signs consistent with HPAI). The Epidemiologic Links/Exposures to Infected Premises section contains questions to help determine whether or not a premises is a Contact Premises (e.g., has the premises been exposed to an HPAI infected flock via poultry manure, poultry carcasses, equipment, etc.). The Biosecurity section helps determine if the premises has a biosecurity program in place that likely will be acceptable to regulatory officials.

**Table 1:** Questions included in the Minnesota Monitored Premises Status Questionnaire (MPSQ).

**IDENTIFICATION AND LOCATION OF PREMISES**
1. What is the national Premises Identification Number (PIN) for the premises?
**HPAI**
**Responses indicate whether or not a premises is an Infected Premises (#2) or a Suspect Premises (#3–5); Yes answers will be referred to Incident Command for follow-up**
2. Does premises have a diagnosis of HPAI? (Yes/No)
3. Does premises have any unexplained clinical signs or clinical signs indicating HPAI? (Yes/No)
4. Does premises have any unexplained mortality or mortality indicating HPAI? (Yes/No)
5. Does premises have any unexplained changes in production parameters or production parameters indicating HPAI? (Yes/No)
**EPIDEMIOLOGIC LINKS/EXPOSURES TO INFECTED PREMISES**
**Responses indicate whether or not a premises is a Contact Premises; Yes and Unknown answers will be referred to Incident Command for follow-up**
6. Has this premises been exposed to poultry manure from an infected flock (HPAI virus in manure) in the past 14 days? (Yes/No/Unknown)
7. Has this premises been exposed to dead poultry from an infected flock (HPAI virus in carcasses, etc.) in the past 14 days? (Yes/No/Unknown)
8. Has this premises been exposed to live poultry from an infected flock (HPAI virus in bird secretions and excretions) in the past 14 days? (Yes/No/Unknown)
9. Has this premises been exposed to eggs or egg-handling materials from an infected flock (HPAI virus in and on eggs from infected birds) in the past 14 days? (Yes/No/Unknown)
10. Has this premises been exposed to semen or semen-handling materials from an infected flock (HPAI virus in semen) in the past 14 days? (Yes/No/Unknown)
11. Has this premises had unmitigated exposure^*^ to equipment that has been in contact with poultry manure, dead poultry, live poultry, eggs, egg-handling materials, semen, or semen-handling materials from an infected flock in the past 14 days? (Yes/No/Unknown)
12. Has this premises had unmitigated exposure^**^ to people who have been in contact with poultry manure, dead poultry, live poultry, eggs or egg handling materials from an infected flock in the past 14 days? (Yes/No/Unknown)
13. Have the people or the equipment from this premises been involved in the depopulation of infected flocks in the past 14 days? (Yes/No/Unknown)
**BIOSECURITY**
**An answer of No will be referred to Incident Command for follow-up**
14. Is an Accredited Veterinarian (or other Biosecurity Coordinator) responsible for the development, implementation, maintenance, and ongoing effectiveness of a premises biosecurity program that conforms to the National Poultry Improvement Plan (NPIP) guidelines? (Yes/No)

*Additional required and critical information also is gathered in the full MPSQ (i.e., date and time that status of Infected Premises was last checked in order to ensure up-to-date Contact Premises information; and explanations of any “yes/no/unknown” answers where appropriate)*.

* *Unmitigated exposure to equipment means inadequate sanitation procedures for those items that come into contact with an infected flock or infectious materials such as trucks/trailers used to transport live birds, eggs, or eggshells; load-out equipment; dumpsters; etc. (a longer list of examples is included with the full MPSQ)*.

** *Unmitigated exposure to people means inadequate biosecurity, sanitation, or downtime procedures for those people who come in contact with an infected flock or infectious materials such as might happen with working at other poultry operations, visiting a poultry processing plant, visiting a manure handling plant, etc. (a longer list of examples is included with the full MPSQ)*.

The questionnaire was designed with the intent that industry representatives, who actually know answers to farm level questions, will initially answer the questions. Gearing the questionnaire toward industry-initiated determination of MP status was an intentional redistribution of permitting-related responsibility based on two lessons learned: (1) determining appropriate premises designation can take more time and poultry commodity expertise than regulatory personnel may have available during an outbreak, and (2) for products that move daily, MP status must be continuously re-evaluated during an outbreak as Infected Premises status (and thus potential Contact Premises) can rapidly change; this amount of work for regulators concerned with outbreak control may not be justified for low-risk products but is desired by producers who want risk for moving product to be as low as possible. Additionally, the MPSQ questions were designed to be cross-sector and appropriate for any poultry/poultry product (e.g., the terms poultry and bird are used throughout rather than specifying sector/bird type). If a premises meets all of the criteria to be designated as an MP, then the request for permitted movement from that premises should be more easily evaluated by regulatory personnel who make the final determination as to whether to designate a premises as an MP, evaluate compliance with product-specific permit guidance criteria, and issue a permit or not. The pre-existing, targeted, cross-sector MPSQ can enable a more efficient evaluation, and re-evaluation if necessary, by both industry and regulators.

Since the development of the MPSQ, multiple HPAI outbreak tabletop exercises have been conducted throughout the US by the SFS team. These exercises have underscored the complexities involved with the permitting process during an FAD response. Indeed, one of the most commonly identified exercise benefits noted by participants has been knowledge gained about COB permitted movement (9). Discussions from these exercises reinforce that the MPSQ is a tool that can streamline the COB SFS permitting process during what can be a chaotic time. Specifically, it provides industry representatives with a “Grab n' Go” list of questions that they can answer before requesting a COB SFS permit; and it allows regulatory officials to assess those answers quickly (i.e., the answer to all of the MPSQ questions, except for the very first and last questions, should be NO otherwise further follow-up is needed by Incident Command) (see Table 1). Ultimately, the MPSQ makes the evaluation process to determine MP status operational rather than theoretical.

## Conclusion

The need for a logistically feasible operational process that could support the efficient and high throughput of COB permits was a driving force for a series of meetings following the 2014–2015 HPAI outbreak in order to improve the permitting process in MN and gather the experiences gained in the largest FAD outbreak in US history. The process undertaken by MN stakeholders has been collaborative, multi-disciplinary, and multi-layered. Ultimately, the lessons learned from the 2014–2015 outbreak and open discussions resulted in the development of an MN MPSQ with the intent that this instrument could be a universal tool for all poultry commodities and eventually all animal agricultural businesses such as beef, dairy, and pork. MP status is central to the assumptions used to determine the risk of commodity movements during an HPAI outbreak. The MPSQ is a tool that helps to further operationalize the process used to determine MP status for the purposes of permitting, and together with other improvements (e.g., Secure Poultry Supply Plan harmonization, electronic forms and web applications, and risk-based permitting approaches), has improved and streamlined the permitting process in MN and could likely be a beneficial tool for other states' response plans as well.

## Author contributions

JU wrote the manuscript. RJ provided valuable background information regarding the entire permitting improvement process undertaken in Minnesota. EL provided helpful research assistance as well as additional background information regarding the cross-sector meetings. RJ, CC, and DH conceived the MPSQ. MK provided poultry industry leadership throughout the permitting improvement process and review of the MPSQ. RJ, MC, TG, DH, and CC provided valuable expertise, feedback, and editing on all topics included in this manuscript. All the authors have read and approved the manuscript.

### Conflict of interest statement

MK is employed by Jennie-O Turkey Store. The remaining authors declare that the research was conducted in the absence of any commercial or financial relationships that could be construed as a potential conflict of interest.

## Footnotes

^1^The Secure Poultry Supply Plan (SPS) is a translation of the science in the Secure Egg (SES), Turkey (STS), and Broiler (SBS) Supply plans into a harmonized permitting approach that can be readily accessed (Grab n' Go) in the event of a disease outbreak such as HPAI (https://securepoultrysupply.umn.edu/).

## Abbreviations

COB: Continuity of Business
FAD: Foreign Animal Disease
HPAI: Highly Pathogenic Avian Influenza
MN: Minnesota
MP: Monitored Premises
MPSQ: Monitored Premises Status Questionnaire
SFS: Secure Food Supply
UMN: University of Minnesota
USDA APHIS: United States Department of Agriculture, Animal and Plant Health Inspection Service.
